# High Intensity Training Is an Effective Modality to Improve Long-Term Disability and Exercise Capacity in Chronic Nonspecific Low Back Pain: A Randomized Controlled Trial

**DOI:** 10.3390/ijerph182010779

**Published:** 2021-10-14

**Authors:** Jonas Verbrugghe, Dominique Hansen, Christophe Demoulin, Jeanine Verbunt, Nathalie Anne Roussel, Annick Timmermans

**Affiliations:** 1 REVAL—Rehabilitation Research Center, Faculty of Rehabilitation Sciences, Hasselt University, 3590 Diepenbeek, Belgium; dominique.hansen@uhasselt.be (D.H.); Annick.Timmermans@uhasselt.be (A.T.); 2 Heart Centre Hasselt, Jessa Hospital, 3500 Hasselt, Belgium; 3 Department of Sport and Rehabilitation Sciences, University of Liege, 4000 Liege, Belgium; christophe.demoulin@uliege.be; 4 Adelante Centre of Expertise in Rehabilitation and Audiology, 6432CC Hoensbroek, The Netherlands; jeanine.verbunt@maastrichtuniversity.nl; 5 Department of Rehabilitation Medicine, Maastricht University, 6211LK Maastricht, The Netherlands; 6 Faculty of Medicine and Health Sciences, University of Antwerp, 2000 Antwerp, Belgium; nathalie.roussel@uantwerpen.be

**Keywords:** chronic low back pain, exercise therapy, high intensity training

## Abstract

Previous research indicates that high intensity training (HIT) is a more effective exercise modality, as opposed to moderate intensity training (MIT), to improve disability and physical performance in persons with chronic nonspecific low back pain (CNSLBP). However, it is unclear how well benefits are maintained after intervention cessation. This study aimed to evaluate the long-term effectiveness of HIT on disability, pain intensity, patient-specific functioning, exercise capacity, and trunk muscle strength, and to compare the long-term effectiveness of HIT with MIT in persons with CNSLBP. Persons with CNSLBP (*n* = 35) who participated in a randomized controlled trial comparing effects of an HIT versus MIT intervention (24 sessions/12 weeks) were included for evaluation at baseline (PRE), directly after (POST), and six months after program finalization (FU) on disability, pain intensity, exercise capacity, patient-specific functioning, and trunk muscle strength. A general linear model was used to evaluate PRE-FU and POST-FU deltas of these outcome measures in each group (time effects) and differences between HIT and MIT (interaction effects). Ultimately, twenty-nine participants (mean age = 44.1 year) were analysed (HIT:16; MIT:13). Six participants were lost to follow-up. At FU, pain intensity, disability, and patient-specific functioning were maintained at the level of POST (which was significant from PRE, *p* < 0.05) in both groups. However, HIT led to a greater conservation of lowered disability and improved exercise capacity when compared with MIT (*p* < 0.05). HIT leads to a greater maintenance of lowered disability and improved exercise capacity when compared to MIT six months after cessation of a 12-week supervised exercise therapy intervention, in persons with CNSLBP.

## 1. Introduction

Chronic nonspecific low back pain (CNSLBP) is a common musculoskeletal disorder affecting many individuals worldwide [1]. It is characterized by fluctuating pain and high levels of functional disability, and consequently has a major impact on activities of daily living, work, and social interactions [2]. As it is thought to have a multi-factorial origin at its base [3], guidelines for CNSLBP highlight the need for a multimodal therapy design [4]. Exercise therapy (ET) is hereby consistently advocated as an important component in management [5,6]. However, while it is presented as the best-evidenced approach, treatment effect sizes in CNSLBP remain only modest [7].

In this regard, a novel ET method, i.e., high intensity training (HIT), has recently been proven to be a feasible and more effective therapy modality than training protocols at moderate intensity in CNSLBP [8,9]. It produces notably greater decreases in functional disability and improves exercise capacity more in the short term [9]. Also, different HIT protocol modalities have been shown to be equally effective to each other in CNSLBP [10]. Indeed, HIT might be better adapted on a physiological level to increase the physical fitness levels in this population [5,11]. Furthermore, these outcomes are in line with studies in other musculoskeletal disorders such as spondyloarthritis or chronic neck pain using various HIT protocols to improve disease specific outcomes such as pain intensity and physical functioning [12,13].

However, CNSLBP by nature often fluctuates over longer periods [14]. As such, recurrences of gradual pain or episodes with increased pain are very common [15,16]. It is thus necessary to obtain a better insight into how exercise-induced benefits directly measured at the cessation of an intervention are retained [17]. While minimal to moderate improvements are observed consistently upon completion of various exercise interventions, these improvements are typically lost over time [18]. Considering this, the ability to maintain the long-term impact of exercise interventions for CNSLBP remains a challenge [18].

The long-term effectiveness of HIT on specific outcomes has been studied in other musculoskeletal populations, such as improving walking speed in persons with knee osteoarthritis and aerobic fitness and functional ability in rheumatoid arthritis [19,20]. However, currently, no data are available on retention effects of HIT on therapy outcomes in rehabilitation of persons with CNSLBP. Because of the better short-term results by HIT on disability and exercise capacity versus MIT, it is expected that HIT leads to a better retaining of these benefits after cessation of intervention in the long-term, when compared with MIT.

Therefore, the aim of this study is (1) to evaluate long-term effectiveness of HIT on disability, pain intensity, patient-specific functioning, exercise capacity, and trunk muscle strength, and (2) to compare long-term effectiveness of HIT with MIT in persons with CNSLBP.

## 2. Materials and Methods

### 2.1. Trial Design

**Figure 1:**
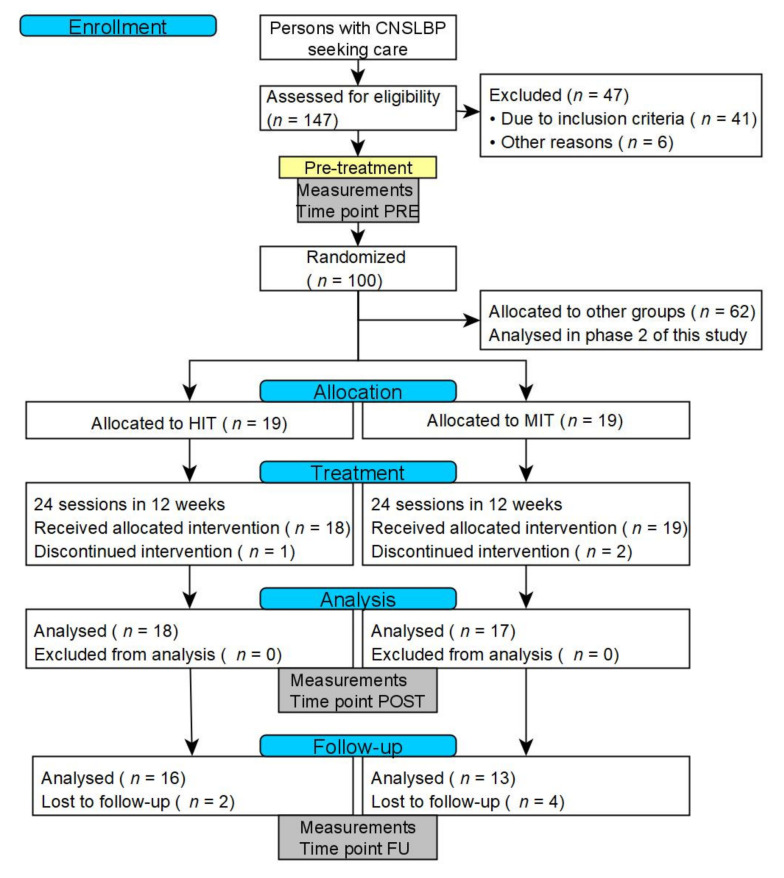
CONSORT flowchart of the research design. Abbreviations: CNSLBP: chronic nonspecific low back pain; HIT: High intensity training; MIT: moderate intensity training.

This exploratory study is part of a larger trial that evaluated the effects of training intensity and training mode in CNSLBP rehabilitation through a prospectively registered, five-arm, RCT organized at REVAL (Hasselt University, Diepenbeek, Belgium). The current article evaluates the effectiveness of HIT in comparison to MIT at six months of follow-up. A comprehensive research design flowchart is displayed in Figure 1. This project was approved by the Medical Ethics Committee of Jessa Hospital (Hasselt, Belgium) and registered at clinicaltrials.gov as NCT02911987.

### 2.2. Participants and Recruitment

Participants were recruited through local study advertisements in Limburg (Belgium). To be eligible, persons had to speak Dutch, be 25–60 years old, and have medically diagnosed CNSLBP [21,22]. Persons were excluded when they had a history of spinal fusion, had a musculoskeletal disorder aside from CNSLBP that could affect the execution of the therapy program, had co-morbidities (e.g., paresis and/or sensory disturbances by neurological causes), were pregnant, had ongoing compensation claims and/or a work disability >six months, had followed an exercise intervention for low back pain in the past three months, or were not able to attend regular therapy appointments. Interested persons received a patient information letter and were invited for an intake session. During that session, the information letter was reviewed, study inclusion and exclusion criteria were evaluated, the informed consent was signed, and a study specific screening form concerning red flags for low back pain rehabilitation was filled out.

### 2.3. Randomization and Blinding

Participants were randomly assigned to an experimental group (‘HIT’) performing a high intensity training program, or a control group (‘MIT’) performing the same training program at moderate intensity. To ensure concealment of allocation, a research assistant not involved in the study picked a sealed, opaque envelope containing the allocated group for each participant. Given the nature of the exercise intervention it was not possible to blind the participants and physiotherapists for group assignment. To limit the performance bias of the participants, the study was described to the participants as ‘a comparison between different modes of exercise therapy treatment’.

### 2.4. Interventions

Participants of both groups were enrolled in a 12-week exercise therapy program consisting of 24 supervised individual therapy sessions (2 × 1.5 h/week). The training protocols have been published more extensively previously [9].

*Experimental group (‘HIT’):* This group performed a protocol consisting of cardiorespiratory training, general resistance training, and core muscle training, all at high intensity (see also Table 1).

Cardiorespiratory training consisted of an interval protocol on a cycle ergometer containing five high intensity one-minute bouts (110 revolutions per minute (RPM) at 100% of the VO_2_ max workload achieved during the maximal cardiopulmonary exercise test, separated by one minute of active recovery (75 RPM at 50% of the same VO_2_ max workload). High intensity bouts increased every two sessions by 10″. Recovery time between bouts remained stable. This protocol was repeated from session 13 to 24 with an updated workload, based on the results from a complementary cardiopulmonary exercise test.

General resistance training consisted of three upper and three lower body exercises executed on fitness devices. A one repetition maximum (1 RM) testing [23] was performed for each exercise. One set of a maximum of twelve repetitions was performed at 80% 1 RM for each exercise. Researchers progressively increased the exercise weight when the participant was able to perform more than 10 repetitions on two consecutive training sessions.

Core strength training consisted of six static core exercises. Exercises were chosen as a function of their ability to load the core muscles at an intensity of at least 40–60% of the maximum voluntary contraction [24]. Participants performed one set of ten repetitions of a ten second static hold. Participants were encouraged to hold the last repetition as long as possible. Exercises were made more difficult by increasing the static hold time and progressing to a more demanding posture when they were executed with a stable core posture for the indicated time by the participant on two consecutive training sessions.

*Control group (‘MIT’):* This group performed a protocol consisting of cardiorespiratory training, general resistance training, and core muscle training, all at moderate intensity (see also Table 1).

Cardiorespiratory training consisted of a continuous training protocol on a cycle ergometer containing 14 min of cycling (90 RPM at 60% VO_2_ max workload). Duration increased every two sessions with 1 min 40 s up to 22 min 40 s. This protocol was repeated from sessions 13 to 24 with an updated workload, extracted from a complementary cardiopulmonary exercise test.

General resistance training was identical to the HIT protocol with the exception of the exercise intensity. One set of 15 repetitions was performed at 60% of 1 RM.

Core training was identical to the HIT protocol with the exception of the exercise intensity. Participants performed one set of 10 repetitions of a 10 s static hold. Exercises were made more difficult when they were executed with a stable core posture for the indicated time by increasing the time of the static hold each six sessions.

### 2.5. Testing Procedure and Outcomes

The following baseline participant characteristics were collected: gender, age (years), weight (kg), and height (cm), to calculate BMI, time of onset of CNSLBP (years, months), fear of movement (Tampa Scale for Kinesiophobia), and physical activity (Physical Activity Scale for Individuals with Physical Disabilities) [25,26]. Outcome measures are described below and were collected at baseline (‘PRE’), at the end of the intervention program (‘POST’), and six months after cessation of the intervention program (‘FU’). At POST, participants were only advised to stay active and were not assisted or tracked in any way. They were not aware they would be invited for a test six months later.

*Disability level—*The Modified Oswestry Disability Index (MODI) evaluates CNSLBP-related disability and consists of 10 items scored on a five-point scale [27]. Total score is expressed in percentage of disability (higher is more) and displays a degree of functional limitation.

*Pain intensity—*The Numeric Pain Rating Score (NPRS) evaluates average pain intensity in the previous six-week period by choosing a number of the 0–10 scale (0 means no pain and 10 means worst pain imaginable) [28].

*Patient-specific functioning*—The Patient-Specific Functioning Scale (PSFS) evaluates individual-specific functioning [29]. Participants state three to five of the most relevant activities compromised due to physical disability and rate them on a 0–10 numeric rating scale (0 means unable to perform and 10 means able to perform at preinjury level). An overall mean percentage is calculated.

*Exercise capacity—*A maximal cardiopulmonary exercise test (75 RPM) on an electronically braked cycle ergometer (eBike Basic, General Electric GmbH, Frankfurt am Main, Germany) evaluates exercise capacity through maximal oxygen uptake (VO_2_ max) and maximal workload through cycling time (min.) [30]. Participants started at a low workload that gradually increased each minute (♂: 30 W + 15 W/min, ♀: 20 W + 10 W/min). Supplementary, respiratory exchange ratio (RER) and heart rate were determined through breath-by-breath gas exchange analysis (MetaMax 3B, Cortex Medical, Leipzig, Germany) and heartrate monitoring (Polar, Kempele, Finland).

*Trunk muscle strength—*A maximal isometric muscle strength test of the trunk flexors and extensors using an isokinetic dynamometer (System 3, Biodex, Enraf-Nonius [31]) evaluates peak torque of trunk flexors and extensors during three maximal repetitions of isometric trunk flexion and trunk extension [32]. Peak torque was expressed in Newton meter (Nm) and normalized to bodyweight (Nm/kg).

### 2.6. Data Analysis

JMP Pro (12.0, SAS Institute Inc., Cary, NC, USA) was used for data analysis. A sample size calculation was performed to detect differences in the primary outcome measure (disability measured by the MODI) between the groups at POST in the primary analysis [9], resulting in a total needed amount of *n* = 34 (*n* = 17 per group). A post-hoc observed power analysis was performed to confirm the specific power for each evaluated outcome measure in the current analysis. Descriptive statistics were used to display baseline group characteristics. Normality and homoscedasticity of each primary outcome were checked by fitting a general linear model of the PRE-FU and POST-FU deltas and plotting the residuals to look for equal variance, symmetry, and identify possible outliers. A general linear model (MANOVA) was used to evaluate the PRE-FU and POST-FU deltas of each outcome measure in each group and the differences between the HIT and MIT group (interaction effect). An alpha level of 0.05 (two-tailed) was used. Percentage improvement of PRE-FU deltas was calculated to evaluate minimal clinically important differences [33]. Regarding the drop-outs, no imputation of data was performed, under the assumption that data were missing at random. However, to check for selective drop-out, differences between participants completing the trial and drop-outs were examined (independent *t*-tests, Mann-Whitney U tests, X^2^ tests).

## 3. Results

### 3.1. Recruitment and Baseline Data

Thirty-eight participants were included in the initial PRE-POST analysis (HIT: *n* = 19, MIT: *n* = 19). Significantly more women (69%) were included. Mean age was 44.1 years (SD = 9.8) and mean pain onset was 11.7 years (SD = 7.7). Both study groups had similar demographics, clinical characteristics, and outcome measures at baseline (*p* > 0.05), except for trunk extensor strength (higher in the HIT group). Nonetheless, all treatment effects were adjusted for these baseline estimates. An overview of the patient characteristics at baseline is displayed in Table 2.

### 3.2. Intervention and Follow-Up Drop-Outs

During the intervention phase, three drop-outs were noted (HIT: *n* = 1, MIT: *n* = 2.8% of all participants from PRE to POST). During the six-month follow-up phase, another six drop-outs (HIT: *n* = 2, MIT: *n* = 4.17% of all participants from POST to FU) were noted. Of these, three participants reported practical issues and three participants did not report any reason for drop-out. No differences in baseline characteristics were found between drop-outs with or without reasons, or drop-outs and other participants. Finally, 29 participants were included in the FU analysis (corresponding to a 24% drop-out in total). No adverse events were noted during this study.

### 3.3. Outcomes at the 6-Month Follow-Up Assessment

An overview of the results is presented in Table 3.

MODI outcomes remained significantly lower compared to PRE in both groups (−13.0 points, 62% improvement in HIT; −5.8 points, 36% improvement in MIT). No significant difference was found from POST to FU in either group. A significant difference of 3.6 points was found in the deltas of PRE to FU between groups.

NPRS outcomes remained significantly lower compared to PRE in both groups (−3.3 points, 59% improvement in HIT; −2.7 points, 54% improvement in MIT). A significant decrease was also found from POST to FU in MIT, but not in HIT. No significant difference was found in the deltas of PRE to FU between groups.

PSFS outcomes remained significantly higher compared to PRE in both groups (+26%, +57% improvement in HIT; +36%, +90% improvement in MIT). No significant difference was found from POST to FU in either group nor in the deltas of PRE to FU between groups.

VO_2_ max remained significantly higher compared to PRE in HIT (3.1 mL/kg/min, 10% improvement), but not in MIT (no improvement at FU). No significant difference was found from POST to FU in either group. A significant difference of 3.2 mL/kg/min was found in the deltas of PRE to FU between groups.

Abdominal muscle strength did not improve compared to PRE in both groups (0.05 Nm/kg, 4% improvement in HIT; 0.03 Nm/kg, 2% improvement in MIT). Back muscle strength remained significantly better compared to PRE in in MIT (0.34 Nm/kg, 13% improvement), but not in HIT (0.19 Nm/kg, 6% improvement). No significant difference was found from POST to FU in either group nor in the deltas of PRE to FU between groups in both outcomes.

## 4. Discussion

This study was the first to evaluate the long-term effects of HIT in CNSLBP. Results show that initial positive therapy effects at the finalization of the therapy program were retained for all outcomes until at least six months later, as no differences could be found between POST and FU results. Furthermore, improvements since baseline on disability level and exercise capacity remained clinically relevant and remained significantly larger in the HIT than in the MIT group at FU [27,34]. These results corroborate the effectiveness of HIT as a working therapeutic modality in the rehabilitation of CNSLBP.

The evaluation of long-term effects of ET studies in CNSLBP has been incorporated in systematic review analyses [5,7]. However, there is still a paucity of pooled data due to heterogeneous ET protocols. Furthermore, FITT-VP principles of exercise prescription (i.e., frequency, intensity, time, and type—volume and progression [35]) are often insufficiently defined, making it even more difficult to evaluate the impact of these program methodology characteristics on therapy success [5]. Only three other studies were found with a clear description of training intensity and a comparison between ET protocols in CNSLBP. Firstly, Michaelson et al. (2016) depicted no differences between a high and low load training program at 12 or 24months follow-up [36]. However, in this article, the magnitude of the load was actually based on an analysis of volume rather than intensity. Besides, an indirect estimation of intensity was made, and no clear objective test was performed to show the actual percentage (e.g., 1 RM testing). Secondly, both Harts et al. (2004) and Helmhout et al. (2008) evaluated the difference between a high and low intensity lumbar extensor program [37,38]. Neither found differences between exercise intensities in the short nor the long term. However, these studies reflected on the use of a very specific strength training mode focused solely on the rationale of restoring back muscle function. Also, training volume was significantly lower, and the high intensity protocols that were used (ranging from 35% 1 RM to max. 70% 1 RM in the HIT group) did not meet the standards used in the present analysis (80% 1 RM strength training).

In the current study, significant differences were noted between PRE and FU in both the experimental HIT and the control MIT group, indicating the effective longevity of ET as a therapy modality. However, no additional improvements from POST to FU were found in either group. This result supports the outcomes of previous research showing that patients who present with low back pain often improve markedly in the first six weeks of rehabilitation therapy. After that, improvement often slows down [18]. This process can even be magnified after cessation of the therapy program. Low to moderate levels of pain and disability are frequently still present at one year after cessation of therapy, especially in the cohorts with persistent pain [39]. It should be noted that the sample in our study already showed low pain intensity (HIT: 2.6/10; MIT: 3.5/10) and disability level (HIT: 7.5/100; MIT: 10.6/100) at POST, which would make further significant improvements very hard to achieve. The only significant difference found from POST to FU was a pain intensity decrease in MIT (3.5/10 to 2.3/10), but not in HIT (2.6/10 to 2.3/10). Thus, while at first glance this might look like an important outcome to support the long-term application of MIT, this difference was actually due to the faster decrease in HIT already achieved at POST (i.e., during the therapy phase). As such, HIT seems to be able to lower pain intensity more quickly. As this was only evaluated with a subjective measure in this study, future research could try to incorporate more objective measures to improve our understanding of pain and pain processing such as pain pressure thresholds through quantitative sensory testing [40,41].

As participants did still display residual pain at FU, adaptations to further optimize the HIT modality should also be investigated. Following current clinical guidelines [6], the authors believe HIT should be incorporated in a multimodal therapy design, as this might stimulate the impact on other factors related to CNSLBP [6]. As such, HIT can be coupled with other important therapy modalities such as delivery of (pain) education and evaluation of and adaptation of therapy to individual therapy goals [42,43]. In addition, further research towards the predictors for therapy success is needed.

### 4.1. Limitations

Limitations of the initial RCT methodology have been discussed previously [9]. Nonetheless, some limitations specifically related to this follow-up analysis should be mentioned. Firstly, because the follow-up analysis was a secondary analysis, study group sample sizes were not initially designed for long-term follow-up. However, even with low power (as measured in a post-hoc analysis), significant results were found in this study, supporting its outcomes. Furthermore, the depicted MODI and VO_2_ max outcomes were still in line with the results from the short-term analysis (that were fully powered). As such, we believe these outcomes to give a fair representation of the expected outcomes in a fully powered sample. Secondly, a follow-up of only six months was performed, which might be low for evaluating the effects of an intervention on long term health behavior. Other research has shown that, up to two years, the same outcomes might be expected but later a regression might occur if behaviors are not changed [44]. However, as this was the first study to evaluate HIT at follow-up, we chose a measurement point at which we expected loss to follow-up would still be manageable (to ensure proper statistical analysis). It is not yet clear whether continuing to perform HIT protocols after a rehabilitation program is needed to retain results beyond six months. Thirdly, physical activity might be a confounder in the maintaining of results during the period between POST and FU. The absence of any longitudinal data related to physical activity performed by the participants might therefore have caused a performance bias when comparing between participants. Indeed, keeping up regular physical activity and adhering to specific exercise programs after the rehabilitation phase have been noted to support therapy success and prevent reoccurrence of chronic low back pain in the long term [45,46]. Besides, multiple psychosocial factors such as perceived stress, self-efficacy, and patients’ perceptions about back pain have also been found to predict development and chronification of low back pain [47,48]. As such, future research should emphasize more on incorporating these factors and evaluating their mediating effects. Fourthly, nine participants dropped out during the course of the protocol from PRE to FU. Results of these persons might have been less favorable. However, no significant differences in baseline characteristics were found between these drop-outs and the included patients. Moreover, no claims with regard to a CNSLBP-related cause to abort the protocol were made by any participant.

### 4.2. Future Recommendations

To be able to provide guidelines, better insights on the working mechanism of this therapy modality are needed. It is still unclear whether HIT improves outcomes due to its increased physical demands and the accompanied physiological factors such as improved muscle characteristics and anti-inflammatory factors, or other non-physiological factors such as increased self-efficacy or fear of movement [49,50].

## 5. Conclusions

High intensity training is an effective therapy modality to decrease disease-specific and physical performance related outcomes in the long term in CNSLBP. Moreover, at six months after cessation, HIT shows greater improvements in disability and exercise capacity than an equal exercise therapy program performed at moderate intensity. Future research is needed to evaluate the exact working mechanisms of this therapy modality and optimize therapy protocols.

## Figures and Tables

**Table 1:** Overview of the content of the intervention program for the experimental (HIT) and control (MIT) group.

Training Modalities	HIT	MIT
Cardiorespiratory protocol	Interval cycling protocol at 100% VO_2_ max	Continuous cycling protocol at 50–60% VO_2_ max
General resistance protocol	Three upper and three lower body exercises at 80% 1 RM	Three upper and three lower body exercises at 60% 1 RM
Core strength protocol	Six static core exercises at an intensity of at least 40–60% MVC until failure	Six static core exercises at an intensity of up to 40% MVC

Abbreviations: VO_2_ max: maximal oxygen uptake; 1 RM: one repetition maximum; MVC: maximum voluntary contraction.

**Table 2:** Demographic and clinical characteristics of participants at baseline (*n* = 38).

Variables	HIT (*n* = 19)	MIT (*n* = 19)	*p*-Value
Gender (m/f)	6/13	6/13	1.000
Age (y)	44.3 (8.8)	44.0 (11.0)	0.769
Symptom duration (y)	11.8 (8.4)	10.3 (7.1)	0.268
BMI (kg/m^2^)	25.6 (4.0)	25.9 (3.6)	0.609
PASIPD, 0–199	16.5 (10.6)	14.9 (11.7)	0.637
TSK, 17–68	32.0 (6.0)	34.7 (7.2)	0.218

Categorical variables are expressed as number, continuous variables are expressed as mean (SD). Abbreviations: m/f: male/female; y: years; kg: kilograms; m: meters; PASIPD: The Physical Activity Scale for Individuals with Physical Disabilities; TSK: Tampa Scale for Kinesiophobia.

**Table 3:** Results of the outcome measures collected from participants at PRE, POST, and FU together with between group differences and post hoc power calculations at FU).

*n* = 29	HIT (*n* = 16)			MIT (*n* = 13)			Interaction at FU (T0-FU)	
Outcome Measures	PRE	POST	FU	PRE	POST	FU	DOD	Power
*Primary*								
Disability								
MODI, %	20.9 (8.7)	7.5 (5.4) *	7.9 (8.4) *	16.2 (8.2)	10.6 (3.0) *	10.4 (9.6) *	3.6 **	0.52
Pain intensity								
NPRS, 0–10	5.6 (1.5)	2.6 (1.3) *	2.3 (2.1) *	5.0 (1.7)	3.5 (1.7) *	2.3 (1.1) *^,†^	0.5	0.09
*Secundary*								
Function								
PSFS, %	46 (18)	71 (15) *	72 (13) *	40 (14)	67 (17) *	76 (15) *	10	0.22
Exercise capacity								
VO_2_ max, mL/kg/min	30.6 (6.8)	35.7 (6.8) *	33.7 (6.5) *	31.6 (7.6)	32.5 (6.3)	31.6 (7.2)	3.2 **	0.61
Relative Muscle strength								
Abdominal, Nm/kg	1.38 (0.28)	1.43 (0.31)	1.43 (0.24)	1.26 (0.37)	1.29 (0.33)	1.29 (0.37)	0.02	0.06
Back, Nm/kg	3.28 (0.82)	3.53 (0.86) *	3.47 (0.84)	2.58 (0.61)	2.87 (0.76) *	2.92 (0.91) *	0.15	0.11

Values in HIT and MIT are reported as mean (standard deviation) and represent results of the Numeric Pain Rating Scale (NPRS), Modified Oswestry Disability Index (MODI), Patient-Specific Functioning Scale (PSFS), a cardiopulmonary exercise capacity test, and a maximum isometric muscle strength test of the abdominals and back, before (PRE) and after (POST) 24 sessions of high intensity training (HIT, 100% VO_2_ max interval cardio training + >80% 1 RM general resistance training + >60% MVC core strength training) or moderate intensity training (MIT, 50–60% VO_2_ max cardio training + 60% 1 RM general resistance training + 20–40% MVC core strength training). Delta displays the post-pre difference. Abbreviations: DOD: difference of deltas of PRE to FU in HIT compared to MIT; CI: 95% confidence interval. * *p* < 0,05 compared to PRE. ^†^ *p* < 0.05 compared to POST. ** *p* < 0.05 HIT compared to MIT.

## Data Availability

The data that support the findings of this study are available on request from the corresponding author [JoV]. The data are not publicly available due to restrictions i.e., their containing information that could compromise the privacy of research participants.
